# Weekly epirubicin plus docetaxel as first-line treatment in metastatic breast cancer

**DOI:** 10.1038/sj.bjc.6603982

**Published:** 2007-10-16

**Authors:** T Gamucci, A M D'Ottavio, E Magnolfi, M Barduagni, A Vaccaro, I Sperduti, L Moscetti, F Belli, L Meliffi

**Affiliations:** 1 Department of Medical Oncology, SS Trinità Hospital, Sora, Italy; 2 Department of Medical Oncology, S Giuseppe Hospital, Albano, Italy; 3 Department of Medical Oncology, Belcolle Hospital, Viterbo, Italy; 4 Department of Medical Oncology, Ospedali Riuniti, Anzio/Nettuno, Italy

**Keywords:** metastatic breast cancer, chemotherapy, weekly epirubicin, weekly docetaxel, weekly schedule, three week schedule

## Abstract

This study was designed to evaluate the efficacy and tolerability of a weekly schedule of epirubicin in combination with docetaxel in the first-line treatment of patients with metastatic breast cancer (MBC). A total of 43 women with MBC not previously treated with chemotherapy for metastatic disease received weekly epirubicin 25 mg m^−2^ and docetaxel 25 mg m^−2^ for a maximum of five cycles (total cumulative epirubicin dose of ⩽900 mg m^−2^). Dose reduction was not permitted. Objective response and evaluation of toxicity profile were the primary study end points; time to progression and overall survival were secondary end points. Patients were followed for a median of 21 (4–38) months. Analysis was by intent to treat; 33 patients completed five cycles of therapy, and the median dose of epirubicin administered to the 43 patients was 23 mg m^−2^. Twenty-five patients (58%) achieved a partial response and one (2%) achieved a complete response. An additional 12 patients (28%) had stable disease. The median time to progression was 11 months (95% confidence intervals (CI) 7–14) overall, and 13 months (95% CI 12–14) in the 26 patients who responded to treatment. Median overall survival was 25 months for responders and 14 months for nonresponders. Grade 3/4 neutropenia occurred in 16% of patients and in 6% of cycles. One patient developed cardiac toxicity (20% reduction in left ventricular ejection fraction). The combination of epirubicin plus docetaxel is highly active in MBC, with a manageable toxicity profile. Such a weekly schedule might provide a valuable treatment option for MBC.

Breast cancer is the most prevalent malignancy globally and the leading cause of cancer-related death in women, with more than a million newly diagnosed cases occurring worldwide annually (Parkin *et al*, 2005). Furthermore, it is estimated that 30–75% of patients undergoing surgery and adjuvant treatment will develop recurrent disease (Paridaens, 2000).

Metastatic breast cancer (MBC) is essentially incurable with standard therapy and patients with MBC have a median survival of about 2 years after metastases have been detected (Henderson, 1991). As a consequence, treatment goals are to improve symptoms, prolong survival, and maintain or improve quality of life. A 5-year survival rate ranging from 10 to 30% has been observed in metastatic disease confined to the bone, which is characterised by an indolent clinical course. However, patients with visceral (mainly liver) metastases have a worse prognosis and a median survival time of approximately 8 months (Piccart, 1996). Thus, treatment end points of MBC are currently improvement of objective response rate (ORR), time to progression (TTP) and overall survival (OS), but the role of chemotherapy in this setting is still unclear.

The taxanes and the anthracyclines are considered among the most active single agents for the first-line treatment of MBC. Consequently, the combined use of taxanes and anthracyclines is a logical step in the search for highly effective chemotherapy combinations. Unfortunately, the clinical utility of anthracyclines is limited by cardiac toxicity. Epirubicin, however, is less cardiotoxic than doxorubicin, showing a toxicity ratio of 1 : 1.8 when compared to the latter, while retaining similar activity and efficacy (Robert, 1993). Furthermore, the semisynthetic taxane docetaxel retains much of its therapeutic activity even in patients unresponsive to anthracyclines (Ten Bokkel-Huinink *et al*, 1994; Ravdin *et al*, 1995; Valero *et al*, 1995). In addition to the high clinical activity of the two drug classes and the lack of complete clinical cross-resistance, docetaxel and epirubicin have largely non-overlapping toxicity profiles and different mechanisms of action (topoisomerase II inhibition *vs* microtubular assembly disturbance). Combination chemotherapy with a 3-week schedule in phase II trials improved response (46–88%) (Mavroudis *et al*, 2000; Pagani *et al*, 2000; Milla-Santos *et al*, 2001; Morales *et al*, 2004) without an associated higher incidence of cardiotoxicity. The high response rates were attained in patients with unfavourable prognostic factors, including multiple metastatic sites, visceral metastases and prior exposure to adjuvant chemotherapy. However, the dose-limiting toxicity for this combination was neutropenia.

A dose–response relationship has been shown for single agent epirubicin in women with MBC (Bastholt *et al*, 1996). Doubling the epirubicin dose from 50 to 100 mg m^−2^ also was shown to significantly increase ORR values in a phase III study in MBC (Brufman *et al*, 1997).

A strategy to reduce toxicity without prophylactic granulocyte colony-stimulating factor (G-CSF) support is to use the combination therapy according to a weekly schedule. A weekly schedule could maintain peak blood concentrations below the threshold value of medullar toxicity. This results in a more favourable safety profile, and facilitates maintenance of the correct dosing schedule and completion of the administration cycles (Norton, 1988, 2005; Burstein *et al*, 2000; Stammler *et al*, 2001; Wenzel *et al*, 2002).

On the basis of these premises, the present study was designed to evaluate the therapeutic efficacy and tolerability of a regimen of weekly epirubicin in combination with docetaxel in the first-line treatment of MBC.

## PATIENTS AND METHODS

### Patient eligibility

The study included women, aged more than 18 years, with a histological or cytological diagnosis of MBC and who had not received previous chemotherapy for metastatic disease. Geographic accessibility for the treatment and follow-up was considered as an inclusion criterion. Previous adjuvant and/or neoadjuvant chemotherapy were allowed provided that they had been completed at least 1 year before enrolment. If patients received anthracyclines as adjuvant treatment, disease progression had to have occurred at least 12 months after completion of anthracycline treatment. The cumulative previous anthracycline dose was not to exceed 240 mg m^−2^ for doxorubicin or 450 mg m^−2^ for epirubicin. Previous hormone adjuvant therapy or treatment for metastatic disease was allowed. Other inclusion criteria were the presence of at least one lesion measurable in two dimensions, World Health Organization performance status 0–2, adequate haematological function (neutrophils ⩾1500 × 10^9^ l^−1^, haemoglobin ⩾10 **g** dl^−1^ and platelets ⩾100 × 10^9^ l), creatinine ⩽140 mmol l^−1^ (1.6 mg dl^−1^), bilirubin ⩽1.5-fold the upper normal limit, aspartate and alanine aminotransferase ⩽1.5 times the upper normal limit, alkaline phosphatase ⩽2.5-fold the upper normal limit (with the exception of bone metastases in the absence of liver metastases), absence of congestive heart failure or angina even if therapeutically controlled, absence of arterial hypertension or arrhythmias not under pharmacological control, absence of myocardial infarction or unstable angina pectoris in the 12 months before the onset of the study, and normal cardiac function evaluated by 12-lead electrocardiogram and echocardiography (normal left ventricular ejection fraction (LVEF ⩾50%)).

Patients were excluded if they had received radiation therapy of the lesion chosen to evaluate response to treatment, with the exception of progressive disease. Radiation therapy must have been completed at least 4 weeks before the study. Bone marrow irradiation of an area equal to 20% was allowed. Patients with symptomatic brain or leptomeningeal metastases, infection, malnutrition or a history of secondary malignant neoplasm other than basal or squamous cell carcinoma of the skin, or carcinoma *in situ* of the uterine neck (adequately treated and not relapsing in the 5 years preceding the study) were not included. Additional exclusion criteria were previous history of neurological or psychiatric diseases, symptomatic peripheral neuropathy above grade 2, severe respiratory failure, uncontrolled diabetes mellitus, acute peptic ulcer or other contraindication to the use of corticosteroid premedication, concomitant treatment with other experimental drugs, concomitant hormone therapy, pregnancy and breastfeeding. The Ethics and Scientific Institutional Review Boards of the participating centres approved the study. All patients signed an informed consent form before entering the study.

### Patient evaluation

Baseline evaluation not exceeding 4 weeks before the first infusion consisted of a complete medical history and physical examination. Serum biochemical profile, electrocardiogram, chest radiograph, abdominal ultrasound and/or computed tomography scan, measurement of carcinoembryonic antigen and CA 15.3 determination, bone scintigraphy and bone X-ray were all performed at the time of enrolment. Appropriate imaging studies to assess objective response (chest X-ray, abdomen ultrasound, CT scan of thorax and abdomen, bone scintigraphy or X-ray) were performed after the 1st, 3rd and 5th cycle of treatment.

### Study design

Patients were treated weekly with epirubicin (Farmorubicine; Pharmacia, Milan, Italy) 25 mg m^−2^ administered as a 15-min intravenous infusion followed 1 h later by docetaxel (Taxotere; Aventis, Antony, France) 25 mg m^−2^ as a 60-min intravenous infusion, according to a schedule of 6 weeks in every 8 for the first cycle then 3 weeks in every 5. Treatment continuation after the first cycle was allowed in the presence of an objective response or stable disease and in the absence of severe toxicity. All patients were given prophylactic corticosteroid premedication to avoid hypersensitivity reactions and to prevent the occurrence of uid retention; this consisted of oral prednisone 25 mg 12 h before and 24 h after treatment, dexamethasone 4 mg and ranitidine 50 mg 30 min before infusion; prophylactic antiemetic treatment consisted of 5-hydroxytryptamine type-3 receptor antagonists administered 30 min before infusion and when needed. A maximum of five cycles were given for a total cumulative epirubicin dose of ⩽900 mg m^−2^ during the study period. No dose reduction was permitted. Treatment was delayed for 1–2 weeks in the event of febrile neutropenia, thrombocytopenia ⩾grade 2, gastrointestinal toxicity ⩾grade 2 and neurological toxicity ⩾grade 2. Epirubicin administration was delayed in patients who developed LVEF ⩽10% of the initial value or below the normal limit.

### Assessment of toxicity

Weekly blood cell count, monthly complete serum biochemistry, electrocardiogram every two cycles, and echocardiography after the 1st and 3rd cycle and at the end of treatment to evaluate LVEF were performed. Toxic effects were graded by the National Cancer Institute Common Toxicity Criteria. Physical evaluation and performance status scoring were also performed at the same time points. An undesired event, whether or not related to the administered drugs, was defined as an adverse reaction. This included any side effect, trauma, toxicity or hypersensitivity reaction, as well as any unexpected clinical or laboratory result. A fatal event, or any event causing life-threatening injury, requiring hospitalisation or producing disability was considered a serious adverse event. Death or congenital anomalies not related to primary disease or drug overdose were always considered as serious events. Worsening of concomitant or predisposing disease was considered as a serious event not related to the trial treatment, or related to treatment failure or early effect.

### Assessment of response

Assessment of antitumour activity was evaluated by the same instrumental approach used to evaluate target lesion(s) at study entry and was scored according to the Response Evaluation Criteria in Solid Tumors (RECIST) Group criteria. Tumour response was based on subsequent assessments performed after the 1st and 3rd cycle and at the end of treatment. Objective responses were graded according to standard criteria for complete or partial response, stable disease, or no change, and progressive disease. The TTP was calculated from the time of the first dose to the time of the first objective evidence of tumour progression. Overall survival was calculated from the time of patient enrolment to the time of documented death. All patients who completed at least the first cycle of therapy were considered eligible for response to treatment evaluation.

### Statistical analysis

The study was designed as a phase II trial. The primary objectives were the evaluation of the therapeutic efficacy (response rate) of combined epirubicin plus docetaxel as a first-line treatment for patients with MBC and the evaluation of the toxicity profile of this combined regimen. Secondary objectives were the evaluation of TTP and OS. All patients included in the trial were evaluated according to intent-to-treat analysis. The sample size necessary for the study was determined using the tables for single-stage phase II trials based on the exact binomial distribution proposed by A’Hern (2001). Considering a response rate of 40% as not acceptable and seeking to evaluate whether treatment would lead to a response rate of 60%, the calculated sample size was 42 patients with a statistical power of 80% using the 5% level of significance. Unless otherwise specified, results are reported as median (range) along with 95% confidence intervals (CI). The Kaplan–Meier method was used to calculate OS and progression-free survival curves, and the significance test was assessed according to the Mantel log-rank test. All calculations were made using computer software packages SPSS 13.0.

## RESULTS

### Patient characteristics

Forty-three patients (median age 59 years, ranging from 36 to 79 years) were enrolled in the study and followed for a median of 21 months (range 4–38). Among the 43 patients, 42 (98%) had a performance status of 0–1, 20 (46.5%) had one site of metastasis and 5 (11.6%) had metastatic involvement of three or more sites. Twenty-four patients (55.8%) had received previous adjuvant chemotherapy, 14 (32.5%) with anthracyclines and 10 (23.3%) with CMF. The baseline characteristics of the patients are listed in Table 1.

### Treatment

Of the 43 patients included in the study, 33 completed five cycles of treatment, whereas 10 were withdrawn due to progressive disease (six patients), adverse events (three patients: two chemotherapy-related and one after a fracture of the femur) or refusal to continue after the first cycle of treatment (one patient). Of the 190 treatment cycles administered (median five cycles per patient, range 1–5), two patients (4.7%) were delayed due to treatment toxicity and eight patients (18.6%) were delayed due to non-drug-related causes. The median duration of treatment delay was 159 days (range 28–203 days). The median dose of epirubicin administered to the 43 patients included in the analysis was 23 (range 17–25) mg m^−2^.

### Adverse events

Toxicities per cycle and per patient are shown in Table 2. When all cycles were considered, grade 3/4 neutropenia occurred in 7 patients (16%) and 11 cycles (6%). Grade 1/2 anaemia occurred in 14 patients (32%), and 4 patients (9%) had thrombocytopenia. The most frequent non-haematological toxicities were seen in four patients (9%) who had grade 3 asthenia and two patients (5%) who developed grade 3 gastrointestinal events (diarrhea in one patient and vomiting in the other). Mild fluid retention or oedema was observed in five patients (12%). The median LVEF was 60% (range 51–79%) at baseline and 62% (range 48–78%) at the end of treatment. One patient had asymptomatic cardiac toxicity (20% reduction in LVEF; from 60 to 48%).

### Efficacy

Twenty-five of the 43 patients enrolled in the study (58.1%) achieved a partial response, and 1 (2.3%) had a complete response, for an ORR of 61% (95% CI 46–75; Table 3). Stable disease was seen in 12 patients (27.9%) and progressive disease in 5 (11.6%) during or after completion of treatment. The ORRs were not influenced by the previous adjuvant treatment. In fact no statistically significant differences were observed in the responses’ distribution in patients who had not received adjuvant chemotherapy, CMF or anthracyclines (RR, respectively, 63.2 *vs* 70 *vs* 50%; *P*=0.58).

**Figure 1:**
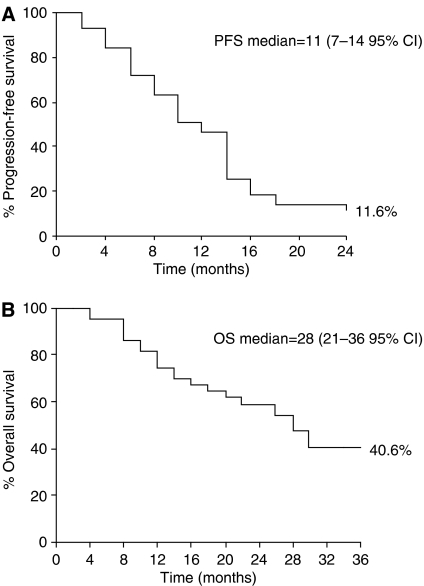
(**A**) Progression-free survival of patients treated with weekly epirubicin in combination with docetaxel. (**B**) Overall survival of patients treated with weekly epirubicin in combination with docetaxel.

With a median follow-up of 21 months, the median TTP was 11 months (95% CI 7–14) and OS 28 months (95% CI 21–36). In 26 patients who responded to treatment, the TTP was 13 months (95% CI 12–14) compared to 7 months (95% CI 2–11) in 17 patients who did not respond. Median OS was 29 months (95% CI 22–35) for responsive patients compared to 20 months (95% CI 16–26) in patients who did not respond to treatment. Progression-free survival and OS over time are presented in Figure 1A and B, respectively.

## DISCUSSION

The combination of anthracyclines and docetaxel has demonstrated significant activity as first-line chemotherapy in MBC. The rationale for combining docetaxel with anthracyclines rests on a number of observations. Individually they have the greatest single-agent activity in patients with advanced breast cancer, lack substantial clinical cross-resistance and have largely non-overlapping patterns of commonly encountered adverse events (Nabholtz, 2003). In particular, increased cardiac toxicity has not been reported in clinical trials. The dose-limiting toxicity for this combination is myelosuppression, specifically a high incidence of grade 4 neutropenia and febrile neutropenia, which is manageable with haematopoietic growth factors (Morales *et al*, 2004). A clear dose–response relationship has been demonstrated for epirubicin and docetaxel as single agents, with or without G-CSF support (Bastholt *et al*, 1996; Trudeau *et al*, 1996; Sparano *et al*, 2000; Milla-Santos *et al*, 2001). However, it was recently reported that dose-escalated epirubicin and docetaxel every 3 weeks, with prophylactic G-CSF, resulted in severe myelosuppression without improving efficacy, compared with other studies of taxane/anthracycline combinations (Fabi *et al*, 2004). Hence, the current study was aimed at evaluating the therapeutic efficacy and tolerability of epirubicin plus docetaxel combination regimen administered on a weekly schedule.

The rational behind this choice was centred on the dose-density theory based on the reduction of the intervals between chemotherapy doses to restrict the opportunity for cancer cells to become resistant to drugs and to target cell clones with differing growth rates. Furthermore, weekly administration of chemotherapy in a dose-dense schedule is understood to have an anti-angiogenesis effect, constricting the blood supply to tumours and restricting their growth. Thus, the weekly schedule was designed to allow administration of a total dose of drug greater than or equal to that administered with the conventional 3-week schedule, to enhance cumulative cytotoxic activity while reducing the toxicity of the treatment, providing greater therapeutic benefit together with a more favourable tolerability profile (Norton, 1988, 2005; Burstein *et al*, 2000; Stammler *et al*, 2001; Wenzel *et al*, 2002).

The ORR achieved with this schedule (60.5% (95% CI 45.9–75.1)) was similar in patients with or without previous chemotherapy, and with or without previous anthracycline exposure. Complete and partial response rates were 2.3 and 58.1%, respectively. The median TTP in patients who responded was 13 months (95% CI 12–14) and the median OS was 29 months (95% CI 22–35). Our findings are similar to those reported for the docetaxel arm in a phase III trial in which doxorubicin (50 mg m^−2^) and docetaxel (75 mg m^−2^) was compared with doxorubicin (60 mg m^−2^) and cyclophosphamide (600 mg m^−2^) for a maximum of eight cycles in 423 patients with MBC (Nabholtz and Riva, 2001). Patients included in the docetaxel arm showed a significantly improved ORR (60%, *P*=0.012) and median TTP (37.1 weeks, *P*=0.0153). In the phase II study performed by Mavroudis *et al* (2000), treatment with 70 mg m^−2^ of epirubicin and 90 mg m^−2^ of docetaxel resulted in an ORR of 66% with a median TTP of 11 months. The combination of docetaxel and epirubicin without G-CSF support resulted in febrile neutropenia in approximately 15% of cycles in a trial by Pagani *et al* (1999) and in 4 and 7% of cycles in the trials by Morales *et al* (2004) and Mavroudis *et al* (2000), respectively. In our study, febrile neutropenia was not observed.

Although the number of patients enrolled was small, this result is very promising, particularly considering that prophylactic G-CSF was not given, and that G-CSF support for the treatment of grade 3/4 neutropenia was given in only 5.8% of cycles. Importantly, cardiac toxicity, as evidenced by reduced LVEF, was observed in only one patient.

The median duration of response (10 months), TTP (11 months) and OS (28 months) in our trial are very acceptable for patients with an incidence of visceral metastases of approximately 65% and a high rate of adjuvant chemotherapy, including anthracyclines. Furthermore, the results are very similar to those found in other studies using a conventional 3-week schedule.

In conclusion, our study confirmed that the combination of docetaxel and epirubicin is highly active in MBC and has a favourable toxicity profile when administered in a weekly schedule, which allowed administration of the full-programmed dosages without the necessity of dosage reduction due to side effects. This is of particular importance to allow the administration of effective regimens providing clinical benefit to an often-elderly population of patients with metastatic disease. The lack of cardiac toxicity of this regimen is also of particular interest because anti-HER 2 therapies, now widely available, may increase the risk of cardiac morbidity in patients heavily pretreated with anthracyclines. Furthermore, compared to standard monochemotherapy treatments with Docetaxel every 3 weeks, this regimen shows to be more effective and less toxic (O’Shaughnessy *et al*, 2002). The combination of epirubicin and docetaxel in a weekly schedule might be considered as an important option in the treatment of MBC.

## Figures and Tables

**Table 1:** Baseline patient characteristics

**Characteristic**	**No.**	**%**
Total	43	100

*Grading*		
G1	4	9.3
G2	13	30.2
G3	15	34.9
Unknown	11	25.6

*Receptor status*		
ER−	12	27.9
ER+	28	65.1
ER Unknown	3	7.0
PR−	14	32.6
PR+	26	60.5
PR unknown	3	7.0
Her-2/neu−	17	39.5
Her-2/neu+	6	14.0
Her-2/neu unknown	20	46.5

*Menopausal status*		
Pre	16	37.2
Post	27	62.8

*Histotype*		
Ductal	37	86.0
Lobular	3	7.0
Other	3	7.0

*Tumour size*		
T1	8	18.6
T2	18	41.9
T3	3	7.0
T4	7	16.3
Unknown	7	16.3

*Nodal status*		
N0	11	25.6
N1	18	41.9
N2	6	14.0
N3	1	2.3
Unknown	7	16.3

*Performance status*		
0	35	81.4
1	7	16.3
2	1	2.3

*Previous adjuvant therapy*		
Chemotherapy		
No	19	44.2
Anthracyclines	14	32.5
CMF	10	23.3

Radiation therapy		
No	26	60.5
Yes	17	39.5

Hormone therapy		
No	19	44.2
Yes	24	55.8

Hormone therapy for metastatic disease		
No	34	79.1
Yes	9	20.9

*Number of metastatic sites*		
1	20	46.5
2	18	41.9
3	5	11.6

*Site of metastases*		
Soft tissues	7	16.3
Visceral	28	65.1
Bone	8	18.6

Abbreviations: CMF, cyclophosphamide plus methotrexate plus fluorouracil; ER, estrogen receptor; PR, progesterone receptor.

**Table 2:** Adverse events per patient and per cycle

	**Patients (%)**				**Cycles (%)**			
	**Grade**				**Grade**			
**Adverse event**	**1**	**2**	**3**	**4**	**1**	**2**	**3**	**4**
Asthenia	23.3	11.6	9.3	0	14.2	3.7	2.1	0
Cardiac	0	2.3	0	0	0	0.5	0	0
Cutaneous	2.3	0	2.3	0	0.5	0	0.5	0
Diarrhea	9.3	7.0	2.3	0	3.2	2.6	0.5	0
Anemia	20.9	11.6	0	0	13.2	4.2	0	0
Hypersensitivity	0	0	0	0	0	0	0	0
Leucopenia	2.3	4.7	0	0	0.5	1.1	0	0
Stomatitis	9.3	11.6	2.3	0	3.2	3.2	0.5	0
Nausea	23.3	4.7	0	0	8.4	1.1	0	0
Neurological	4.7	0	0	0	2.1	0	0	0
Neutropenia	14.0	9.3	11.6	4.7	7.4	5.8	4.2	1.6
Thrombocytopenia	9.3	0	0	2.3	4.2	0	0	0.5
Fluid retention	7.0	4.7	0	0	3.2	2.1	0	0
Vomiting	14.0	4.7	2.3	0	6.3	2.1	0.5	0

**Table 3:** OS and progression-free survival for patients treated with weekly epirubicin in combination with docetaxel

*Survival (%)*	
1-year	74.4
2-year	59.1
3-year	40.6

Median overall survival, months (95% CI)	28 (21–36)

*Progression-free survival (%)*	
1-year	46.5
2-year	11.6

Median time to progression, months (95% CI)	11 (7–14)

Abbreviations: CI, confidence intervals; OS, overall survival.
